# Thermal dose and radiation dose comparison based on cell survival

**DOI:** 10.1186/2050-5736-3-S1-P26

**Published:** 2015-06-30

**Authors:** Meredith Lee, David Schlesinger, Gail ter Haar, Benjamin Sela, Matt Eames, John Snell, Arik Hananel, Neal Kassell, Jason Sheehan, James Larner, Jean-Francois Aubry

**Affiliations:** 1 Focused Ultrasound Foundation, Charlottesville, Virginia, United States; 2 University of Virginia, Charlottesville, Virginia, United States; 3 The Institute of Cancer Research, London, United Kingdom; 4 Institut Langevin, Paris, France

## Background/introduction

The biologic dose response curves of thermal dose and absorbed radiation dose have not been compared to each other even though they have both been extensively investigated separately and combined. Although heat and radiation produce cell kill by different biological mechanisms (Thermal dose denatures proteins and the radiation dose causes DNA damage) a comparison of dose response curves is possible using the endpoint of cell survival.

## Methods

Survival curves for both thermal and radiation doses were extracted for three different types of cells from previously published data. Using models based on the beam shapes of the current clinical systems for the dose profile, the survival curves were generated and the survival profiles were compared for both modalities, Focused Ultrasound (FUS) and Gamma Knife (GK), for a thalamotomy. The thermal dose profile was calculated according to Dewey (1994), from temperature maps simulated with a 3D finite differences time domain code solving the bio-heat equation with a heat deposition term dependent on the pressure field. Radiosurgery dose distributions were exported from the Gamma Knife treatment planning software (Leksell GammaPlan versions 8.0 - 10.1, Elekta AB, Stockholm) with the smallest target as an input.

## Results and conclusions

**Figure 1:**
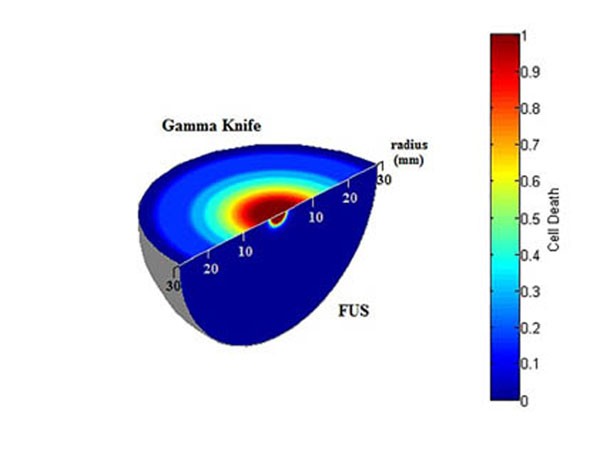
Fraction of cell death radially from the focus of the gamma beams and focused ultrasound beams.

The comparison showed that focused ultrasound exhibits a steeper dose and survival profile than gamma knife. As shown in Figure 1, a smaller percentage of cells are dead a short distance away from the FUS target compared with GK. Also, cell death drops more gradually for GK than FUS. Our results establish that the penumbra is steeper for FUS than GK and have implications for making treatment decisions as well as for rationally combing the two modalities.
